# Decay of HCoV-OC43 infectivity is lower in cell debris-containing media than in fresh culture media

**DOI:** 10.17912/micropub.biology.001092

**Published:** 2024-02-16

**Authors:** Julia Hillung, J. Tomás Lázaro, Juan-Carlos Muñoz-Sánchez, María-José Olmo-Uceda, Josep Sardanyés, Santiago F. Elena

**Affiliations:** 1 Evolutionary Systems Virology, Instituto de Biología Integrativa de Sistemas (I2SysBio), CSIC - Universitat de València, Paterna, 46980 València, Spain.; 2 Dynamical Systems and Computational Virology, CSIC Associated Unit CRM - I2SysBio, Spain; 3 Departament de Matemàtiques, Universitat Politècnica de Catalunya (UPC), 08028 Barcelona, Spain; 4 Institute of Mathematics, UPC - BarcelonaTech (IMTech), 08028 Barcelona, Spain.; 5 Centre de Recerca Matemàtica (CRM), Campus de Bellaterra, Cerdanyola del Vallès, 08193 Barcelona, Spain.; 6 Dynamical Systems and Computational Virology, CSIC Associated Unit CRM - I2SysBio, Spain.; 7 Santa Fe Institute, Santa Fe, New Mexico, United States

## Abstract

In the quantitative description of viral dynamics within cell cultures and, more broadly, in modeling within-host viral infections, a question that commonly arises is whether the degradation of a fraction of the virus could be disregarded in comparison with the massive synthesis of new viral particles. Surprisingly, quantitative data on the synthesis and degradation rates of RNA viruses in cell cultures are scarce. In this study, we investigated the decay of the human betacoronavirus OC43 (HCoV-OC43) infectivity in cell culture lysates and in fresh media. Our findings revealed a significantly slower viral decay rate in the medium containing lysate cells compared to the fresh medium. This observation suggests that the presence of cellular debris from lysed cells may offer protection or stabilize virions, slowing down their degradation. Moreover, the growth rate of HCoV-OC43 infectivity is significantly higher than degradation as long as there are productive cells in the medium, suggesting that, as a first approximation, degradation can be neglected during early infection.

**Figure 1. Growth and decay rates of infectivity for HCoV-OC43:**
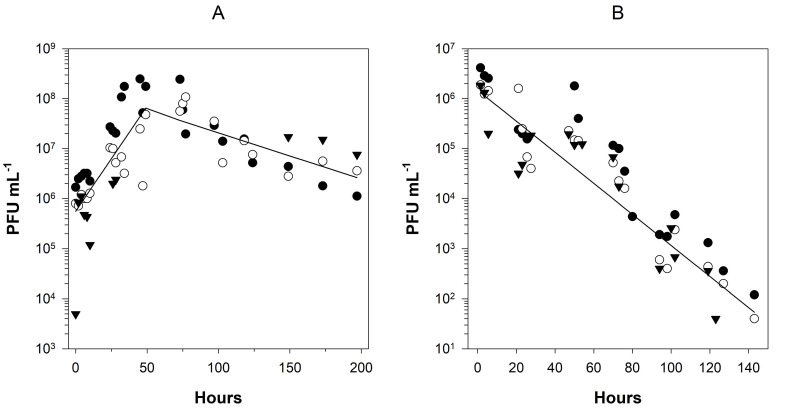
**(A)** Change in HCoV-OC43 infectivity (PFU mL ^-1^ ) in BHK-21 cell cultures along time in logarithm scales. The solid line represents the fit to the biphasic linear model shown in Eq. 1. **(B)** HCoV-OC43 infectivity decay in complete fresh media. The solid line represents the fit to a simple linear model. In both panels, the three experimental replicates are represented by different symbols.

## Description

The most widely used method to prepare fresh stocks of infectious virus particles has been to inoculate them into susceptible cells and allow the virus to replicate and accumulate until no more cells are available for subsequent infections. Depending on the time elapsed between virus production and collection and preservation, degradation may become an important process, reducing the amount of infectious viral particles produced, resulting in a reduction of the specific infectivity of the stock, *e.g* ., measured as the concentration of plaque forming units per unit of media volume (PFU mL ^-1^ ). Despite its practical value, very few quantitative estimates of infectivity decay, or even its value relative to infectivity growth, are available in the literature for a comprehensive set of viruses. For example, Guillier et al. (2020) meta-analysis showed that coronavirus’ decay rate in liquid media depended on temperature and, for example at 20 ºC, 304 h were necessary to reduce titer 10 ^5^ -fold. Joiner et al. (2021) examined the durability of haemorrhagic septicaemia virus and infectious haematopoietic necrosis virus in different substrates, including clay, metal, cell culture media, and filtered and non-filtered river water. Decay rates for both viruses largely varied among substrates, with temperature also having a very significant effect. Interestingly, viral persistence in filtered river water was significantly longer than in unfiltered water, suggesting that in more natural conditions, virus survival would be jeopardized by the presence of all sort of biotic and/or abiotic elements in water. In sharp contrast, Gundy et al. (2009) observed that coronaviruses were faster degradated in filtered tap water compared to unfiltered tap water. They hypothesized that the presence of organic matter in unfiltered water could potentially offer protection to viruses adhering to these organic particles and to suspended solids. Furthermore, from a theoretical and modeling perspective, having accurate measurements of the rates of production and degradation of infectious viral particles is especially important when parameterizing quantitative models brought forward to describe the within-host dynamics and evolution of viral populations ( *e.g* ., Gilchrist et al. 2004; Sardanyés et al. 2009; Pearson et al. 2011) or their evolution and diversification in cell cultures (Cuevas et al. 2005; Martínez et al. 2011; Sardanyés et al. 2012) and tissues (Sardanyés et al. 2008; Sardanyés et al. 2012).

In this study, we inoculated BHK-21 cell confluent monolayers with HCoV-OC43 to estimate the temporal dynamics of virus’ infectivity at 33 ºC. By extending sampling times beyond the end of the growth phase, we have been able of precisely quantify decay rate in media containing cellular debris. For the sake of comparison, we have also measured the decay rate of the virus in fresh complete media in absence of susceptible cells.

In the first set of experiments, we followed the change in infectivity during a complete cycle of infection ( Fig. 1A ). Three independent experimental blocks, each one at a different multiplicity of infection (MOI) were performed (see Methods). After discarding a significant block effect in both growth and decay rates (ANOVAs: *F*_2,32_ = 1.058, *P* = 0.359 and *F*_2,15_ = 1.897, *P* = 0.184, respectively), data were pooled into a single regression analysis. Log-transformed data were fitted to the biphasic linear model described in Eq. 1 ( *R* ^2^ = 0.604, *F*_4,55_ = 2186.571, *P* < 0.001). During the first phase, viral growth was obviously the dominant process. Infectivity increased exponentially at a rate of *g* = 0.0421 ±0.0052 h ^-1^ until reaching a maximum value at *T* = 59.072 ±10.211 h post-inoculation, time at which no more alive cells were observed for additional infections. Afterwards, virus decay was the dominant process, with an estimated rate of infectivity decay of *d* = -0.0092 ±0.0028 h ^-1^ . Therefore, the accumulation of infectious particles was 4.56-fold faster than their degradation.

In a second set of experiments, we evaluated the infectivity decay rate in complete fresh media ( Fig. 1B ), in three independent experimental blocks each with slightly different inoculum size (see Methods). Firstly, we ruled out a potential block effect (ANOVA: *F*_2,53_ = 0.540, *P* = 0.586) and then proceeded to estimate the rate of infectivity decay in this simple environment. In this case, log-transformed data were fitted to a linear regression model, resulting in an estimated decay rate of *d* = -0.0310 ±0.0037 h ^-1^ ( *R* ^2^ = 0.549, *F*_1,57_ = 69.510, *P* < 0.001).

Interestingly, the loss of infectivity in cell-free media was 3.35-fold faster compared to that in a media containing cell lysates, being the difference between both conditions highly significant ( *t*_78_ = 32.925, *P* < 0.001). To explain this difference, we propose two non-mutually exclusive hypotheses. Firstly, being HCoV-OC43 an enveloped virus sensitive to temperature, detergent and other chemical agents (Gundy et al., 2009; Firquet et al., 2015) , it experiences a form of protection from degradation by cellular membranes, even in the lysed medium. Secondly, viruses in cells have undergone new rounds of replication and potentially formed microvesicles before starting the process of degradation. These microvesicles could protect them from degradation. Supporting this hypothesis, it has been shown that SARS-CoV-2 infection alters lipid metabolism and utilize small extracellular vesicles to protect against the attack of the host’s immune system (Maeda et al. 1999; Rosell et al. 2021; Shariq et al. 2023; Xiao et al. 2023; Zavan et al. 2023) . In contrast, viruses that have been directly introduced into the fresh medium after thawing from -80 ºC storage, would be more susceptible to degradation owing to the lack of vesicular protection. If true, this suggests that the protection afforded by vesicles formed by multiple viruses is stronger than toxic factors present in a medium containing cell debris and degradation enzymes, which might destabilize virions.

## Methods

*Cell culture, media and virus*

Baby hamster kidney cells (BHK-21) were generously provided by Dr. Rafael Sanjuán (I2SysBio, CSIC-UV). Prior to infection, cells were cultured at 37 °C in DMEM (GIBCO-ThermoFisher Scientific, Waltham MA, USA), supplemented with 0.22% (w/v) sodium bicarbonate (Sigma-Aldrich, Burlington MA, USA), sodium pyruvate (Sigma-Aldrich), 10% fetal bovine serum (FBS) (GIBCO-ThermoFisher Scientific), 1× penicillin-streptomycin (GIBCO-ThermoFisher Scientific), 1× amphotericin B (GIBCO-ThermoFisher Scientific), and non-essential amino acids, following standard laboratory procedures. The cells were routinely tested and found negative for mycoplasma contamination.

The virus used in this study was HCoV-OC43 (ATCC ^®^ VR-1558). The maintenance medium of cells infected with HCoV-OC43 consisted of the same supplemented DMEM but now with only 2% FBS.

For decay experiments without cells, the cell-free medium used was identical to the maintenance medium for infected BHK-21 cells.

*Infection of BHK-21 cells*

BHK-21 cells were plated in 6-well culture dishes one day prior to infection, reaching 85 - 95% confluency. The cell layers were infected with the virus at MOIs of 3.8, 1.8 and 0.06, each of these conditions representing an experimental block. The cell supernatant was aspirated, and cells were washed once with 1× phosphate buffered saline (PBS). Subsequently, 250 µL of the viral inoculum was added to the cell monolayer, and the mixture was incubated at 33 ºC and 5% CO _2_ with sporadic shaking to prevent cell layer drying. Two hours post-inoculation, 2 mL of maintenance medium were added to the infected cells. HCoV-OC43 was cultured and maintained in the plates with cells and later cell debris for 8 days at 33 ºC. Every 2 h, excluding nighttime, 30 µL of supernatant from infected cells were collected and frozen for later quantification of the viral infectivity.

*Initiation of viral decay experiment in fresh media*

For the viral decay experiment in cells-free fresh media, 250 µL of the virus was added to a 6-well culture plate and incubated at 33 ºC. The dish was sporadically shaken to reproduce the experiment with cells. After 1.5 h, additional 2 mL of maintenance medium were added to the well. At this point, the viral titers were 4.16×10 ^6^ *,* 1 *.* 88×10 ^6^ and 1.92×10 ^6^ PFU mL ^-1^ , each of these conditions represents an experimental block. The culture dishes with HCoV-OC43 were maintained for 144 h at 33 ºC. Every 2 h, excluding nighttime, 30 µL of supernatant were collected and frozen for later quantification of the viral titer.

*Viral titer (infectivity) quantification*

To determine the number of infectious HCoV-OC43 particles at each sampled time point, a plaque assay was performed using BHK-21 cells. The procedure involved seeding the cells into 6-well plates and incubating them for 24 h until reaching 80 - 90 % confluency. Serial dilutions of the virus were prepared in DMEM, and 250 μL of each dilution was used to infect the wells of the 6-well plate for 90 min at 33 ºC and 5% CO _2_ with swinging every 15 min. A negative control consisting of only DMEM without virus was included for comparison. After the infection period, the cells were covered with 2 mL of media, which consisted of a 50:50 mix of 2× DMEM medium supplemented with 2% FBS and 2% agar. The agar-DMEM layer was overlaid with liquid DMEM supplemented with 1% FBS as it solidified. The plates were then incubated at 33 ºC and 5% CO _2_ for 120 h. After incubation, cells in the plates were fixed using 10% formaldehyde and plugs were removed. The cells were then stained with 2% crystal violet in 10% formaldehyde, and plaques were counted to determine the number of PFU per mL of the corresponding inoculum.

*Statistical analyses*

Prior to estimating the kinetic parameters of interest, block effects were evaluated fitting data to random factors model II ANOVA with experimental block and time as orthogonal factors. Type III sum of squares were used. PFU mL ^-1^ data were log-transformed to achieve normality.

The calculation of decay rate in fresh media was performed by directly calculating the linear regression coefficient of log-transformed viral titer ( *V* ) with time ( *t* ): log *V* ( *t* ) = log *V* (0) + *g t* , where *d* stands for the decay rate. By contrast, the infectivity data collected along the entire infection cycle in presence of BHK-21 cells were used for calculating both the growth, *g* , and decay rates by fitting the following biphasic linear model:

log *V* ( *t* ) = log *V* (0) + *g t* – δ *_tT_* *d t* , (1)

where δ is the Kronecker delta function that takes value δ = 0 if *t* < *T* and 1 otherwise, being *T* , a parameter also to be estimated, the inflexion time at which cells are exhausted, viral replication stops and degradation becomes the dominant process. Models were fitted using the Levenberg-Marquardt nonlinear regression method as implemented in SPSS version 29.0.0.0 (IBM, Armonk NY, USA).
